# Electronic communication of cells with a surface mediated by boronic acid saccharide interactions†
†Electronic supplementary information (ESI) available: Experimental detail and supporting figures. See DOI: 10.1039/c5cc04311e



**DOI:** 10.1039/c5cc04311e

**Published:** 2015-09-28

**Authors:** Alex Stephenson-Brown, Sue Yong, Muhammad H. Mansor, Zarrar Hussein, Nga-Chi Yip, Paula M. Mendes, John S. Fossey, Frankie J. Rawson

**Affiliations:** a School of Chemical Engineering , University of Birmingham , Edgbaston , Birmingham , West Midlands B15 2TT , UK; b School of Pharmacy , University of Nottingham , University Park Nottingham , Nottingham , Nottinghamshire , NG7 2RD , UK; c School of Chemistry , University of Birmingham , Edgbaston , Birmingham , West Midlands B15 2TT , UK; d Laboratory of Biophysics and Surface Analysis , School of Pharmacy , University of Nottingham , University Park Nottingham , Nottingham , Nottinghamshire , NG7 2RD , UK . Email: frankie.rawson@nottingham.ac.uk

## Abstract

The fabrication of a molecularly tailored surface functionalised with a saccharide binding motif, a phenyl boronic acid derivative is reported. The functionalised surface facilitated the transfer of electrons, *via* unique electronic interactions mediated by the presence of the boronic acid, from a macrophage cell line. This is the first example of eukaryotic cellular-electrical communication mediated by the binding of cells *via* their cell–surface saccharide units.

## Abstract 2

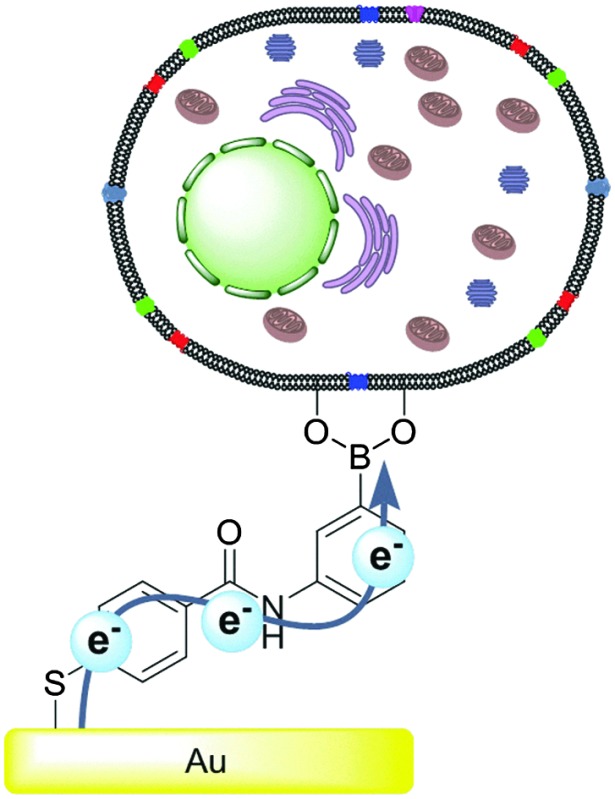
Gold surfaces were molecularly tailored with a saccharide binding motif capable of covalently adhering cells. This facilitated communication *via* the macrophage membrane with implications for understanding mammalian cell signalling.

The development and innovation of technology capable of forming a biocompatible interface between cells and materials in their environment is of great significance for an array of applications, from utilization as a research tool to inform biological investigations through to cell culture to tissue engineering.^1^ One area which has received broad interest in recent years is the development of technologies able to facilitate the sensing of bioelectricity generated by cells.^2^ The majority of previous work has focused on understanding why bacteria transfer electrons to their surrounding environment and the elucidating mechanisms that underpin this.^3^ Bacterial mechanisms of external electron transfer have been manipulated to increase the power output of microbial fuel cells. Bacteria have been genetically modified^4^ to increase the external electron transfer pathways.^5^ Surfaces have been modified with mediators and nanostructures to increase the efficiency of electron transfer from cells, *via* the cell wall, to the electrode surface (defined as electrically wiring of cells).^6,7^ Importantly, such studies provide deeper understanding of how these biochemical pathways, that involve external electron transfer, control cellular function.^8^ To date, a considerable volume of work has been devoted to electrochemical behaviors of prokaryotic organisms.^2,5,9–11^ In contrast, only limited attention has been paid to developing strategies to understand and harness electronic interactions with eukaryotic cells.^7,12^ Addressing this will lead to new insights into the biological role that electronic interactions underpin. The lack of progress in this area may be explained by physiological hurdles posed by eukaryotic organisms. The catabolic system in eukaryotes is principally located in mitochondrial internal membranes,^13^ which act to shield them from direct electrode contact. In contrast, bacterial cells' catabolic respiratory machinery is located in the plasma membrane and is responsible for a majority of external electron transfer. This enables electrodes to have easy access to prokaryote cellular components, such as cytochromes,^14^ capable of expelling electrons directly to an electrode.

However, eukaryotic cells are not electrochemically silent with respect to external electron transfer. It should be noted that all eukaryotic cells have transplasma membrane electron transport systems (tPMETs) within the plasma membrane.^15^ It is only *via* these tPMETs that eukaryotes are known to be capable of expelling electrons directly to the external environment.^15^ Importantly, there are no known reported examples of mammalian cells electrically communicating with the external environment directly from the cell membrane. Therefore, utilisation of surface chemistries capable of electronically interacting with cells may provide new avenues of specifically sensing cellular electrical events and allow access of the biological generated electron pool. This will provide new insight into external electron transfer and how this behaviour can control cell function. Engineering of surfaces capable of electrochemical communication with cells may also allow for the modulation and control of cellular chemistry, and may find application in development of novel electroceutics.^16^ Additionally, such technologies may help accelerate the development of new cellular based fuel cells and provide a deeper understanding of the mechanistic control of external electron transfer sites in eukaryotes. It will provide the opportunity to elucidate how cells electrically communicate and sense their local environment. Herein we report the observation of electronic communication exhibited *via* the plasma membrane of eukaryotic cell, a macrophage. This was achieved *via* the molecular tailoring of surfaces with a boronic acid derivative, a phenomenon which, to the best of our knowledge, has not previously been reported. Boronic acid (BA) modified surfaces were created in order to bind and “wire” cells since surfaces modified with saccharide binding boronic acid motifs have previously been shown to bind saccharides in a variety of applications.^17–22^

Crucially, cell surfaces contain saccharide groups in the form of glycolipids and glycoproteins.^1^ It has previously been demonstrated that bacteria can be anchored by their surface sugar groups to electrodes in a fuel cell, *via* boronic acids which increased the power output.^23^ Thus it was hypothesised that boronic acids could be used as a route to anchor eukaryotic cells, *via* the cell surface saccharides, to a conducting surface which would facilitate, and probe, electron transfer from the cell to the surface and *vice versa*.^24^

It was our intention to test the hypothesis that boronic acids are capable of mediating charge transfer *via* cell surface saccharide receptors.^25^ Since eukaryotic cell surfaces contain glycolipids and glycoproteins, the boronic acid mediated wiring approach, proposed herein, appeared to be an ideal strategy for facilitating electrochemical communication with mammalian cells. Gold surfaces were modified with boronic acids, as depicted in Fig. 1. Surface modifications were confirmed by ellipsometry, contact angle and attenuated total reflectance Fourier transform infrared spectroscopy experiments (see ESI†).

**Fig. 1:**
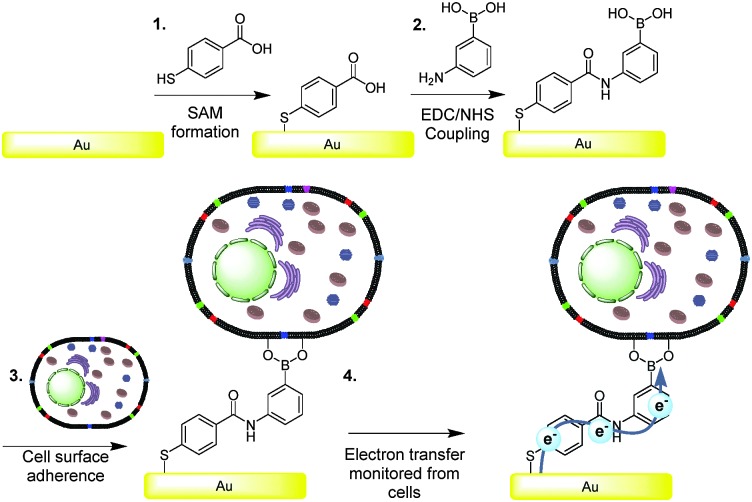
Schematic representation of (1) the formation of the carboxylic acid-terminated SAM, (2) the coupling of 3-aminophenylboronic acid and (3) the binding of cells *via* cyclic boronic ester formation. (4) Charge transfer events from cells enabled by the fabrication of electrodes with boronic acid (BA)-terminated self-assembled monolayers (SAMs).

Experiments were performed by surface plasmon resonance (SPR) spectroscopy to establish the adhesions behaviour of the COOH-SAM (gold modified with SAM) *vs.* the BA-SAM (surfaces modified with SAM then functionalised with boronic acid) surface. The SPR curves (Fig. S2, ESI†) showed that the kinetics of the binding of the macrophage cells was different between the COOH-SAM and BA modified surface, and the number of cells adhered after 40 minutes is different, which was established *via* microscopy cell density studies (see ESI† for details). Interestingly, when cells were exposed to surfaces for a longer time at higher concentrations there was no significant difference between the amount of cells adhering on the COOH-SAM compared to the BA-SAM, with an average number of cells on the COOH-SAM of 1052 mm^–2^ (±1 SD of the mean 147 *n* = 6) and BA-SAM 1068 mm^–2^ (±1 SD of the mean 283 cells *n* = 6) obtained. The early difference in cell numbers attached at the surfaces occurs because of the inherent property of covalent *versus* hydrogen and electrostatic interactions (BA *versus* COOH-SAM, respectively) of the cells to the surface. In order to make comparative electrochemical interrogation studies, and to discount any electrochemical differences that was observed in redox behaviour occurred due to cell density differences, both substrates were left for the longer period so that BA and COOH-SAM surfaces would have a similar number of cells adhered on them and it was on these surfaces that cyclic voltammetry was performed.

Cyclic voltammograms were logged at BA-modified surface, which were incubated in a 15 cm^2^ petri dish in the presence and absence of an addition of 2 × 10^6^ cells (Fig. 2I (green) and II (blue), respectively). A COOH-SAM control surface was also exposed to 2 × 10^6^ cells to elucidate the role of the covalent binding potential of the boronic acid coated surface *versus* the carboxylic acid surface. BA-modified surfaces exposed to cells displayed distinct electron transfer events with the cells. The electrochemical characteristics of this redox behaviour observed at BA modified surface, resulting from the cell–surface communication, led to a reduction peak at –48 mV (P1), an oxidation peaks at 56 mV (P2) (observed at faster scan rates (Fig. 2II)) and an oxidation peak at 150 mV (P3) observed in Fig. 2I. Importantly, no such redox behaviour was seen in the absence of cells. Control experiments using COOH-SAM surfaces that were exposed to cells had no apparent redox peaks (Fig. 2I (red)). These data confirm the requirement of boronic acid in facilitating the observed electron transfer events, and cement boronic acids as the communication anchor of choice in investigating mammalian cell electronic communication phenomena. Cells observed in microscopy images (Fig. 2) were stained with the live stain calcein green. No significant difference in cell viability was observed when a comparison of adhered cells at the COOH-SAM and BA-modified surfaces is made. When the BA-modified surfaces were exposed to fewer cells (5000) the peak currents reduced significantly (Fig. S3B in the ESI†). Thus, the electrochemical signal is proportional to the amount of cells that are exposed to the BA-modified surfaces. Confirmation that the monolayers were stable during the time frame of these experiments was established by performing thiol desorption studies (Fig. S7, ESI†).

**Fig. 2:**
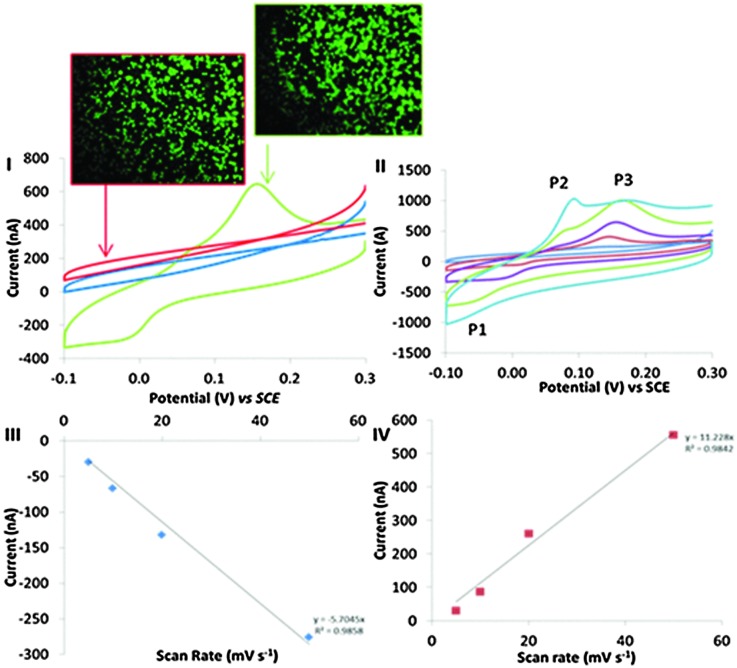
(I) Cyclic voltammograms recorded in 50 mM PBS at electrodes consisting of gold modified with COOH-SAM (red) and 3-aminophenylboronic acid in the presence of cells (green) and electrodes consisting of 3-aminophenylboronic acid which were not exposed to (blue) macrophage cells at 100 mV s^–1^. (II) Cyclic voltammograms recorded at scan rates of 5, 10, 20, 50, 100, mV s^–1^ in PBS on electrodes modified with 3-aminophenylboronic acid and cells. (III) Plots of reductive peak currents (P1) *versus* scan rate. (IV) Plots of oxidative peak currents (PIII) at varying scan rates. Microscopy images of calcein stained cells adhered to COOH-SAM and BA-SAM obtained from modified surfaces exposed to cells for 3 hours confirming similar cell densities.

Evidence that the redox behaviour observed in Fig. 2I originates between the cell–BA-SAM surface interface will now be presented. Peak currents associated with P1 and P3 were shown to be proportional to scan rate, a phenomena indicative of a surface confined process (Fig. 2III and IV).^26^ If the redox signal observed in the cyclic voltammetric studies in the presence of cells was occurring due to a redox active molecule in solution released by the cells during the time frame of the cyclic voltammetric analysis, then the peak currents would be proportional to the square root of scan rate which is indicative of a diffusion controlled process. One interpretation of the observed non-diffusion controlled redox behaviour, observed in the cyclic voltammetry, is that this is because the electron transfer occurs directly between the surface of the cell and the boronic acid attached to the electrode. It does not involve a cell exudate that behaves as an electron shuttle. However, another possible explanation is that an electrochemically active molecule may be released from the cells, which adsorbs to the BA-SAM surface, prior to the electrochemical analysis being performed. To confirm that this was not the case, cells were harvested and cell solutions were filter sterilised with a 0.22 μm filter thereby removing the cells as they are approximately 10 μm in size. The resulting filtrate was appraised electrochemically by performing cyclic voltammetry at BA-SAM surface (Fig. S6, ESI†). Crucially, no discernible redox behaviour was observed. If the species giving rise to the observed behaviour was a molecule that the cells excreted it would be expected that we would observe similar redox behaviour as obtained in the presence of adsorbed cells (Fig. 2I) with the filtered culture medium. On the contrary this was not the case. Moreover, by careful experimental design we have negated the possibility that the observed cyclic voltammetric behaviour was occurring due to dilution effects that occur when cells are trapped at surfaces and release material (see ESI† for detailed discussion). Additionally, It is well known that one function of macrophage in defending against infection is to increase oxidative stress *via* increasing reactive oxygen species (ROS) production.^27^ We have also reasoned that the origin of the redox signal has nothing to do with reactive oxygen species generated that are sequestered by the cells (see ESI† for detailed discussion). All of the above information combined suggests that the electron transfer originates at the cell–BA-SAM surface interface.

An electrochemical mechanistic diagnostic plot was ascertained by comparing the rate at which the voltammetric waves of P1 and P3 shift with scan rate. This plot showed that the redox peaks obtained are typical of an electrochemical system with a CE (chemical step followed by an electrochemical step) and EC mechanism, respectively (Fig. S5, ESI†).^28^ Thus, electrochemical communication through boronic acid mediated covalent recognition of cells displaying surface saccharides is demonstrated. The oxidation event associated with P2 only becomes apparent at faster scan rates, suggesting the chemical step (Fig. 2II) is fast and thus is not observed at slower scan rates and therefore P3 is only observed. A control experiment was performed to confirm that the observed faradaic electrochemistry was occurring due to the unique electronic interaction of the BA-SAM with the cells, and not as a result of saccharide containing groups found within the culture medium. COOH- or BA-SAMs were exposed to modified culture medium in the absence of cells and then cyclic voltammograms were recorded in PBS. No redox peaks were observed (Fig. S5 in ESI†) confirming the voltammetric peaks seen occur due to a cellular mediated process.

Boronic acids have been heavily studied for their ability to bind saccharides selectively and most recently we have shown that they can be used to selective bind and sense glycoproteins *via* saccharide boron interactions in complex media as they bind to sugars preferentially.^29^ The whole rational of using boronic acid modified surfaces to bind cells is based on this premise of them binding sugars found on the cell's surface. Importantly, there have now been a number of examples from other groups which have modified surfaces with boronic acids to anchor cells.^22,23,30^ Matsumoto *et al.* ^30^ showed conclusively that red blood cells could bind to boronic acid modified gold surfaces *via* covalently binding cell surface bound sialic acid. Additionally, they noted that upon binding of erythrocytes *via* cell surface sialic–boron interactions the conductance of the surface changed. Marken *et al.* ^31^ demonstrated that a monomeric acid, caffeic acid, displayed unique redox behaviour, which in part was mediated by their fluxional interaction with boronic acids. Consequently, these literature reports coupled with our experimental observations provide strong evidence to hypothesise that the electrochemistry is coming from cell surface glycan chains and possibly directly from the sialic acid terminus. These acid moieties are commonly found on the termini of glycan chains of the cell surface.^30^ This leads to two possible origins of electron transfers either directly *via* glycoproteins that behave as enzymes due to favourable stereochemistry on binding to the surface. Alternatively, electrons are transferred direct from redox active saccharides bound on the cell surface.^24,32^ However, the biological effect in the changing of the redox state of saccharides on the cell surface is not presently clear and this work demonstrates that this is an area that requires major attention. This will therefore allow for the elucidation of the role that cell surface bound saccharide redox chemistry plays in electronic interactions on the cell with its surroundings. The expulsion of electrons to the external environment in eukaryotic cells has been linked to tPMETs and cellular metabolism. For example, external electron transfer from cells *via* a tPMET based on ferri-reductase, which reduces extracellular iron from Fe^3+^ to Fe^2+^, enables cells to transport iron across the membrane.^33^ With this observation in mind, evidence that the enhancement in current occurred as a result of the biologically generated electrical current was sought. Cells were exposed to a well-known toxin,^34^ 10% ethanol, and continual cyclic voltammograms were recorded. Ethanol causes toxicity by interfering with the membrane integrity. With increasing cycle-number, the peak currents decrease as depicted in Fig. S8 (ESI†). There are two possible explanations for this behaviour: one could be that ethanol causes a cessation of metabolism leading to the cells producing fewer electrons to transfer. The second is ethanol competes in binding with the boron. This means the boronic acid-mediated cellular electronic interaction does not occur. However, both hypotheses further indicate that the origin of the electron transfer seen in the voltammograms is from the interaction of the facade of the cells with the molecularly engineered surface. The importance of this discovery can be placed in context when we consider the known functional role of carbohydrates found on the cell surfaces. One of which is that carbohydrates are known to be involved in cell–cell interactions.^35^

Alongside the demonstrated discovery that mammalian cells are capable of electronically communicating with the external environment, facilitated by cell surface saccharides, we also have previously demonstrated for the first time that electron transfer from inside of yeast cells could occur to the external environment *via* the yeast cell wall, which is largely constituted of polysaccharides.^6^ Consequently, these findings coupled together indicate that cells may communicate through surface–surface saccharide redox chemistry, which opens the door to new interpretation and tools to probe this newly emerging cellular electronic sensing mechanism.

In conclusion, functionalisation of gold surfaces with saccharide binding SAMs has enabled efficient surface adherence of immune cells. This efficient adherence has led to the detection of unique electrical communication with the cells external matrix. Linking cells with a boronate ester linkage provides a new strategy to facilitate charge transfer from eukaryotic cells and to enable real time cell–surface communication. It is envisaged that this platform could be tailored to facilitate communication *via* specific components of the cell membranes, thus providing a new tool for sensing and investigating the role of cellular surface–electronic interactions. In turn, such capabilities are expected to enable new means in which we may be able to control the cell function by using these signalling pathways to direct cellular chemistry.

We would like to thank the Leverhulme Trust (ECF/2013-603), Royal Society (Industry Fellowship), the EPSRC (EP/J003220/1 and EP/K027263/1), the Royal Society of Chemistry (Analytical Trust Fund) ACSS 14/004 and ERC (Consolidator Grant 614787) for funding. We would also like to thank Prof Frank Marken, University of Bath (UK) for helpful suggestions and comments during the preparation of the manuscript.
