# Supplementary material for: Electronic communication of cells with a surface mediated by boronic acid saccharide interactions
Source: Chem Commun (Camb). 2015 Sep 28;51(97):17213–6. doi: 10.1039/c5cc04311e (PMC4668958; doi:10.1039/c5cc04311e)
Supplement: Supplementary file 1 [file CC-051-C5CC04311E-s001.pdf]

Electronic Supplementary Material (ESI) for Chemical Communications.
This journal is © The Royal Society of Chemistry 2015

Electronic Communication of Cells with a Surface Mediated by Boronic Acid Saccharide Interactions

Alex Stephenson-Brown,[^] Sue Yong,[£] Muhammad H. Mansor,[£] Zarrar Hussein,[^] Nga-Chi Yip,[^] John S. Fossey,[$] Paula M. Mendes,[^] Frankie J. Rawson,[£]*

[£]Laboratory of Biophysics and Surface Analysis, School of Pharmacy, University of Nottingham, University Park Nottingham, Nottingham, Nottinghamshire, NG7 2RD, UK.

^School of Chemical Engineering, University of Birmingham, Edgbaston, Birmingham, West Midlands, B15 2TT, UK.

[$]School of Chemistry, University of Birmingham, Edgbaston, Birmingham, West Midlands B15 2TT, UK.

*corresponding author: email: frankie.rawson@nottingham.ac.uk

1. Experimental methods

Contact angle: Contact angle apparatus, equipped with a charged coupled device (CCD) KP-M1E/K camera (Hitachi), was attached to a personal computer for video capture. Dynamic contact angles were recorded as a micro-syringe was used to quasi-statically add water to or remove water from the drop place on the chip. The drop monitored as a live video image, acquisition rate 4 frames per second. FTA (First Ten Angstroms) Video Analysis software v1.96 was used for the analysis of the contact angle of a droplet of water (UHP) at the three-phase intersection. The averages and standard errors of contact angles were determined from five different measurements made for surfaces modified with the self-assembled monolayer (SAM) only and those further modified with the boronic acid SAM.

Ellipsometry: A Jobin-Yvon UVISEL ellipsometer with a xenon light source was used for the measurements. The angle of incidence was fixed at 70°. A wavelength range of 280–820 nm was used. The DeltaPsi software package was employed to determine the thickness of SAMs and SAMs functionalized with 3-aminophenylboronic acid and values and the calculations were based on a three-phase ambient/SAM/Au model, in which the SAM was assumed to be isotropic and assigned a refractive index of 1.50. The thickness reported is the average and standard deviation of six measurements taken on each SAM.

Surface preparation: Polycrystalline gold substrates were purchased from George Albert PVD., Germany and consisted of 50 nm gold layers deposited onto a glass covered with a thin layer of chromium. The gold substrates were cleaned by immersion in piranha solution (7:3, H_2SO_4:H_2O_2) at room temperature for 10 min (Caution: Piranha solution reacts violently with all organic compounds and should be handled with care). Samples removed from the piranha solution were rinsed with water (Ultra High Purity - UHP), followed by ethanol (Fischer Scientific, HPLC grade) for 1 min. Immediately after cleaning, the substrates were immersed in freshly prepared SAM forming solution, 4-mercaptobenzoic acid (1 mM in ethanol (HPLC grade)). Post-immersion in the SAM forming solution, the substrates were rinsed with 2% triethylamine solution followed by ethanol, twice. 3-Aminophenyl boronic acid was coupled to the carboxylic acid-terminated SAMs (COOH-SAM) by first submerging the COOH-SAM-modified surface samples in a 40 mM aqueous solution of 1-ethyl-3-(3-dimethyl aminopropyl) carbodiimide hydrochloride (EDC) and 10 mM N-hydroxysuccinamide (NHS) solution for 1 h and then transferred to a 2 mM solution of 3-aminophenyl boronic acid in phosphate buffer and methanol (7:3 ratio). The electrodes were allowed to react for 24 h before being removed and washed with ethanol and Milli-Q water. The surfaces were characterized with ellipsometry, contact angle and Attenuated Total Reflectance Fourier Transform Infrared Spectroscopy (ATR-FTIR) and detailed description of the methods used can be seen in the supplementary information.

Cell culture: RAW 264.7 (ATCC Lot 59239593) cells were seeded in six 25 cm^2 flask containing 5 ml DMEM (Dulbecco's Modified Eagle Medium modified with foetal bovine serum/HEPES (4-(2-hydroxyethyl)-1-piperazineethanesulfonic acid) and penicillin/streptomycin at 10% FBS, 1% of Penicillin/streptomycin, 2.4% glutamate and 2.4% HEPES). These cells were grown for 2 days at 37 °C in a 5% CO_2 atmosphere reaching an approximate confluence of 80%. The DMEM was aspirated off and 3 mL of fresh DMEM was placed in each flask and cells were detached using a cell scraper. The cells were then counted using a haemocytometer and diluted to give a cell suspension of 2 x 10^6 cells/mL. Prior to assay cells were centrifuged at 1000 rpm and the DMEM aspirated off leaving a cell pellet. The pellet was re-suspended in 50 mM PBS containing 100 mM KCl to give an equivalent cell concentration.

Electrochemistry: Cellular electrochemical studies were carried out with a Gamry 600 potentiostat and data acquisition software (Gamry electrochemistry software version 5.61a), and thiol desorption studies were carried out with a Versastat 4 potentiostat. A three-electrode cell consisting of a saturated calomel reference electrode, Pt counter electrode, and then the working electrode of either Au modified with self-assembled monolayer of (SAM) 4-mercaptobenzoic acid or Au modified with the SAM monolayer which then has 3- aminophenylboronic acid covalently tethered. The electrochemical area was controlled via use of O-rings with fixed diameters. Cyclic voltammetry was performed to determine if direct electrical contact could be made from a starting potential of 0.3 V and a switching potential of −0.1 V and an end potential of 0.3 V at Au modified with SAM and surfaces further modified with the boronic acid in the presence of cells. Cells were adhered to surface by incubating the surfaces with 2×10^6 cells for 3 hours prior to performing cyclic voltammetry. Controls surfaces exposed to the equivalent conditions but without cell exposure were performed. All cyclic

voltammograms were recorded in PBS at 100 mV s^{-1} unless otherwise stated. Linear sweep voltammetry was performed to investigate the thiol reductive desorption. A starting potential of -0.3 V and an end potential of -1.5 V at a scan rate 100 mV s^{-1} was used. The electrolyte was a 0.1 M KOH solution prepared fresh each day. The voltammograms were performed in triplicate The geometric area was controlled by use of a 5 mm diameter O-ring.

Surface Plasmon Resonance (SPR) and Electrochemistry: SPR coupled electrochemical experiments were performed with a Reichert SR7000DC Dual Channel Spectrometer (Buffalo, NY, USA) at 25 °C using a three-electrode electrochemical cell and a Gamry PCI4/G300 potentiostat. A surface modified with SAMs only and 3-aminophenylboronic acid was prepared on Reichert Au sensor chips serving as the working electrode, the counter electrode was a Pt wire, and a SCE was used as the reference electrode. Prior to the cell binding studies, the sensor chips were equilibrated with degassed phosphate buffered saline (PBS), passing degassed PBS through the electrochemical cell at a flow rate of 50 µL min^{-1} for a few hundred seconds. A 5 µL injection of cells containing 5000 cells was injected over the sensor chip surface. CVs were performed at the end of the experiment and the flow rate was switched off to establish if any electron transfer could be ascertained.

Microscopy: All images were taken with Axiolab equipped with an Axiocam CM1 camera for image capture utilising ZEN 2012 Blue edition software by Carl Zeiss Microscopy. Surfaces modified with COOH-SAMs only and 3-aminophenylboronic acid were prepared on Reichert Au sensor chips serving as the working electrode, the counter electrode was a Pt wire, and a SCE was used as the reference electrode. Prior to the cell binding studies, the sensor chips were equilibrated with degassed phosphate buffered saline (PBS), passing degassed PBS through the electrochemical cell at a flow rate of 50 µL min^{-1} for approximately a few hundred seconds. A 5 µL injection of PBS cell suspension containing 5000 cells (dilution from a stock solution of 1×10^6 cells/mL, determined using a heamocytometer) was injected over the sensor chip surface for 40 minutes. The chips were then removed and rinsed in PBS to remove loosely bound cells and then incubated in a PBS solution containing the live cell stain calcein at a concentration of 10 µg/ml for 30 minutes. For longer term cell studies, chips fabricated from polycrystalline gold substrates purchased from George Albert PVD were incubated in a 15 cm^2 petri dish containing 3 ml of modified DMEM and 2 $x10^6$ cells. These were then incubated for 3 hrs at 37°C in 5% CO_2 buffered atmosphere. The chips were removed and gently washed in PBS to remove loosely bound cells. Each chip was then placed into a well of a 12 well plate containing 1 ml of PBS per well. Chips were exposed to 10 µg/ml calcein and incubated for 30 minutes and then images were taken.

ATR-FTIR: The spectra were recorded at a resolution of 16 cm^{-1} with a spectral range of 650-4000 cm^{-1}.The absorption spectra were the result of 64 scans and were recorded at room temperature. The spectra are then recorded using MicroLab FTIR software in a HP laptop connected to the spectrophotometer.

2. Results and Discussion

Gold surfaces were modified with carboxyl acid-terminated SAMs (COOH-SAMs) and then subsequently functionalised with 3-aminophenyl boronic acid (BA-SAMs). To confirm these modifications water contact angle and ellipsometry measurements were performed on the COOH-SAMs and BA-SAMs (Table 1). The advancing contact angles for COOH-SAMs and BA-SAMs are in good agreement with the literature.[1],[2] Previous literature reported a contact angle of 31.5° for a surface modified with boronic acid-terminated SAM that is in good agreement with the value obtained for our BA-SAM, 38.9°. The ellipsometric thickness of the COOH-SAM was determined to be 0.69nm, which is similar to theoretical values in Table 1 and literature values recorded by Barriet *et al*[1]. Following modification with 3-aminophenylboronic acid, the thickness increased to 1.08 nm. The increase in surface thickness of 0.67 nm to 1.08 nm on coupling of the boronic acid to the SAM is significantly different (a paired t-test, p-value < 0.01) confirming the surface modification. To gather more evidence of surface functionalization of the COOH-SAM with BA-modified surfaces, ATR-FTIR spectroscopy was carried out. In Figure 1S, the presence of B-O peak (1342cm^{-1})[3] shown for BA-modified surfaces was present but absent from COOH-SAM, indicating the presence of the BA.

Table 1. Mean advancing and receding water contact angles and ellipsometric thickness for the COOH-SAM with BA-modified surface (Error represents ±1 Standard deviation of the mean n=5). The theoretical molecular lengths were derived from ChemBio3D Ultra 12.0 in which the molecules were in fully extended conformations.

**SAM**	**Contact Angle (°)**		**Thickness (nm)**	
	Advancing	*Receding*	*Theoretical*	*Experimental*
COOH-SAM	18.19±3.3	12.13±2.2	0.67	0.69 ±0.34
BA-SAM	38.9 ±6.1	30.1± 5.1	1.29	1.08 ± 0.27

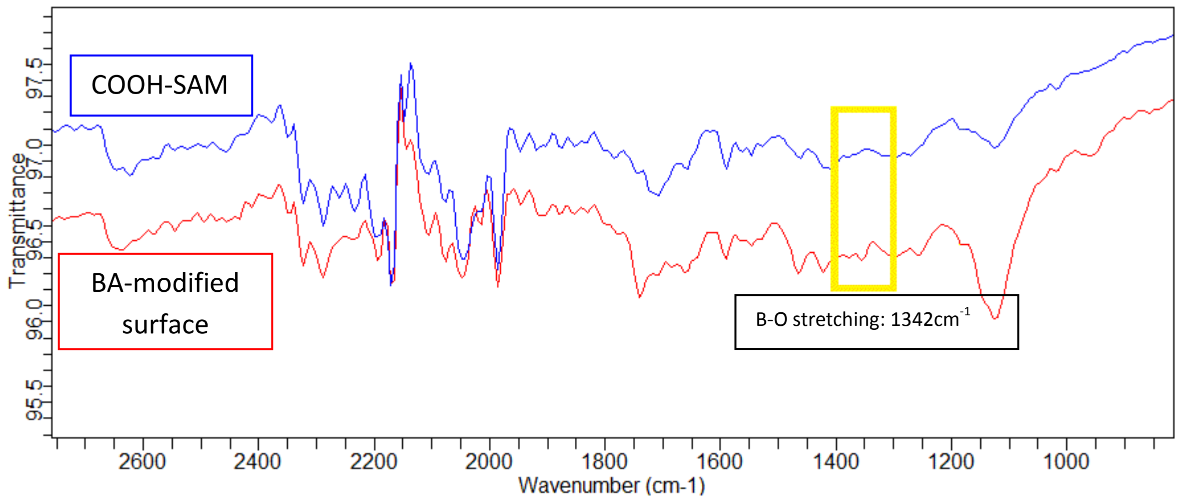

Figure 1S. ATR-FTIR spectra obtained at surfaces modified with COOH-SAM (Blue) and BA-modified surface (Red).

To investigate the role of boronic acid in binding of the cells to the surface, a real-time adherence study was performed using surface plasmon resonance (SPR) spectroscopy with a carboxylic acid (COOH)-SAM *versus* those displaying boronic acids (BA)-SAM. COOH-SAM and BA-modified gold surfaces were exposed to a flow of phosphate buffer saline (PBS) solution pH 7.3 until a steady state was reached. Once a steady state was ascertained, an injection of 5000 macrophage cells at a flow rate of 50 µL min^{-1} was made. As shown in Figure 2S, BA-SAMs elicited significantly higher SPR binding curves than the corresponding COOH-SAM. This behaviour can be attributed to covalent boronic ester formation which leads to increased early cell adhesion to BA modified surfaces when compared to the COOH-SAM surface. The amount of binding in the presence of cells (approximately 2500 RU) superficially appears quite small. For instance, a monolayer of a 20 kD protein would produce a response of similar magnitude. However, previous work using SPR to monitor bacterial cell adhesion to surfaces has produced SPR shifts of comparable amounts[4]. Larger binding responses are not observed in the presence of cells, as the SPR signal is only measured up to 200 nm from the surface meaning that the instrument is only sensitive to the cellular material at the periphery of the cell. As this material is similar in composition to the buffer, being mostly composed of water and salts, the shifts in SPR responses are unsurprising. Consequently, the binding values obtained only represent the cell surface-BA interaction and not the whole cell and explains why the binding units is in an equivalent range of large proteins. The SPR data was also confirmed by performing fluorescent microscopy and analysing the number of cells adhered on COOH-SAM and BA-SAM during SPR studies. A significant difference was observed between the amount of cells adhering on the COOH-SAM compared to the BA-SAM, with an average number of cells adhered on the COOH-SAM of 7 (±1SD of the mean 2 cells n=6) and BA-SAM 48 per mm^2 (± 1SD of the mean 23 cells n=6) obtained (Figure 2S).

We can rule out that the redox behaviour observed in Fig 2 arises due to dilution effects, which may occur when cells are trapped on surfaces and release molecules, as with just hundreds of cells on the surface we observe redox behaviour in the cyclic voltammograms (Fig 3S). It would be envisaged that

with cells adhered at surface compared to an equal number of cells in solutions in which the filtrate would be appraised, the concentration of any excreted molecules at the surface would be significantly higher than the filtrate. Consequently, with the filtrate a false negative may be possible. However, in our studies to ensure this was not the case we used an large number of cells (20 × 10^6) compared to the hundreds for surface studies (Figure 3S) , and the increased length of time in filtrate studies to 48 hours incubation compared to 3 hours for surface studies. Therefore, dilution effects would be negligible and data obtained from cyclic voltammograms suggests that the electrochemical observations of direct electron transfer proceed *via* the cell surface. Additionally, It is well known that one function of macrophage in defending against infection is to increase oxidative stress via increasing reactive oxygen species (ROS) production[5], [6], [7]. ROS have been shown to interact with boronic acids by converting them to phenol which, is an irreversible process [8]. If this were happening then we could expect a reduction in binding as only H-bonding would be possible as seen similarly with the COOH-SAM surface. However, one can note from the SPR results (Figure 2S) for cells adhering to boronic acid surfaces that no decrease in binding is observed. Additionally the process is observed to be chemically reversible as demonstrated by the redox couple in the CV. Therefore, if ROS were converting the boronic acid to phenol which is irreversible, the redox couple in the cyclic voltammograms would not be reversible. Consequently we can therefore eliminate that the origin of the signal has anything to do with reactive oxygen species that are sequestered by the cell

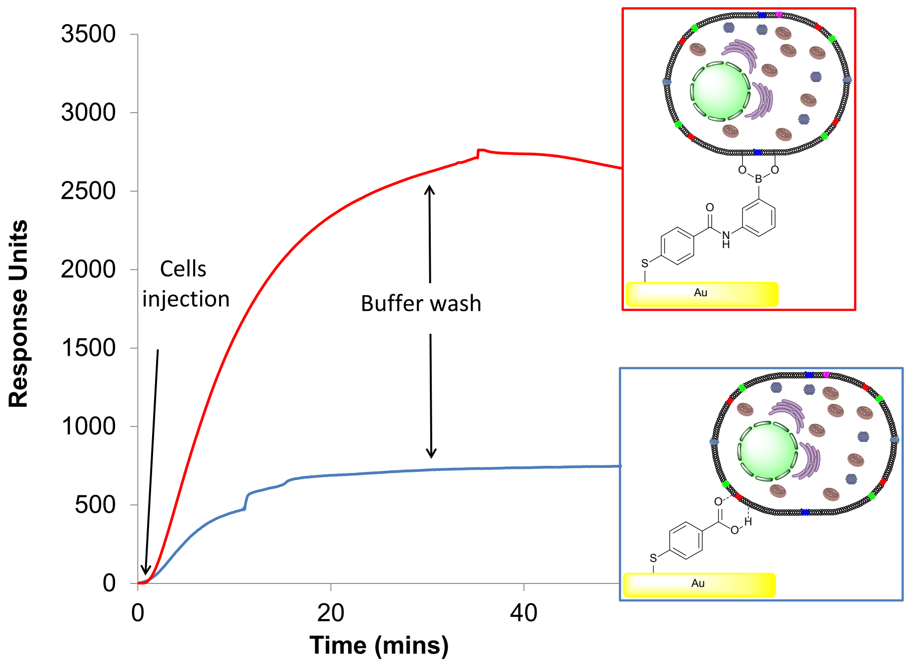

Figure 2S. SPR sensorgrams showing the binding of Raw 264.7 cells at gold surfaces modified with COOH-SAM (Blue) and further modified with 3-aminophenylboronic acid (Red).

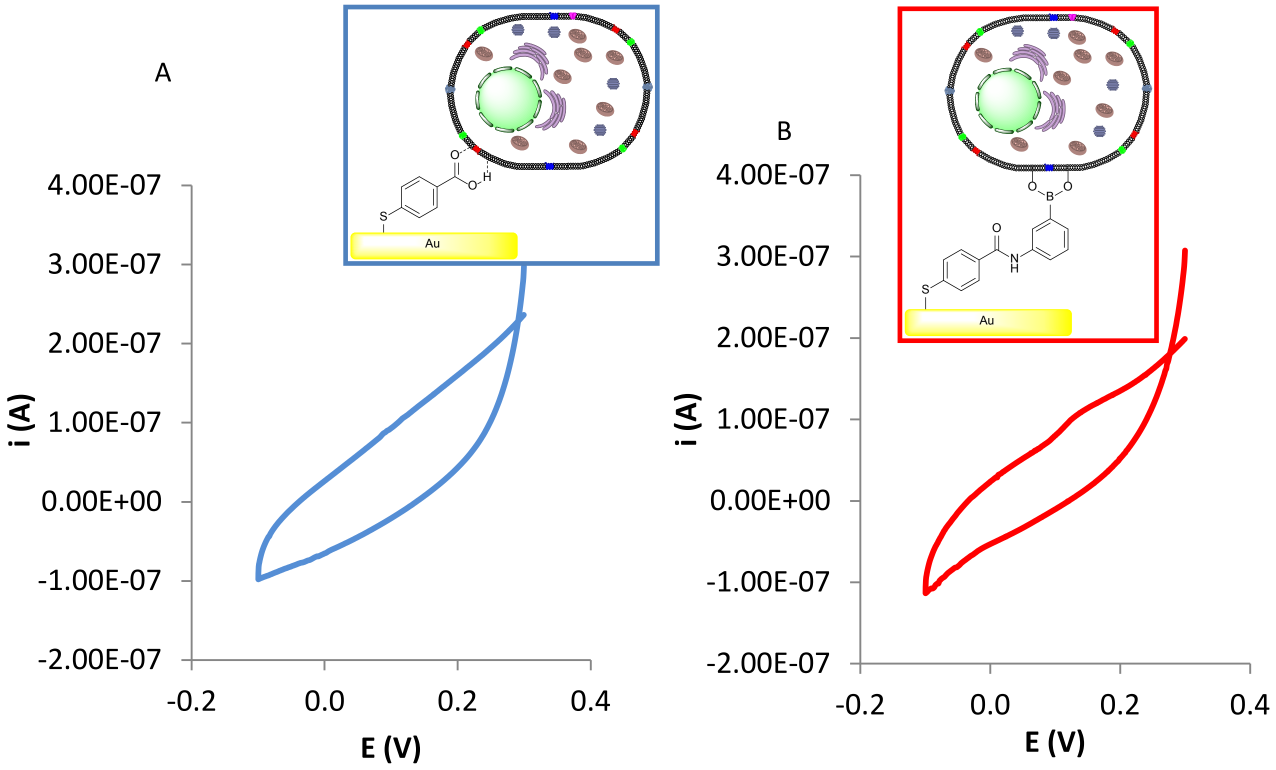

Figure 3S. *In-situ* cyclic voltammograms logged after recording of SPR sensorgrams (Fig 2) showing the binding of Raw 264.7 cells at gold surfaces modified with COOH-SAM (A) and further modified with 3-aminophenylboronic acid (B) at 100 mV s^{-1}.

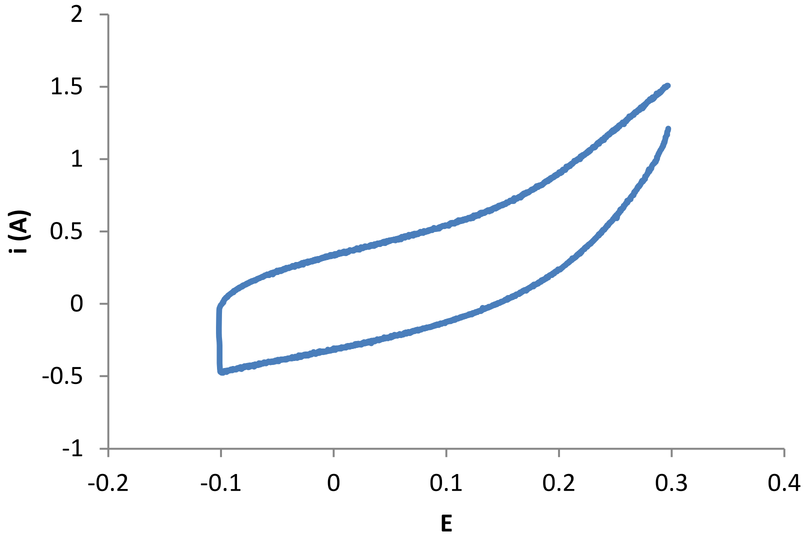

Figure 4S. Typical cylic voltammogram logged at boronic acid modified surface exposed to filtered culture medium used to incubate cells at 100 mV s^{-1}.

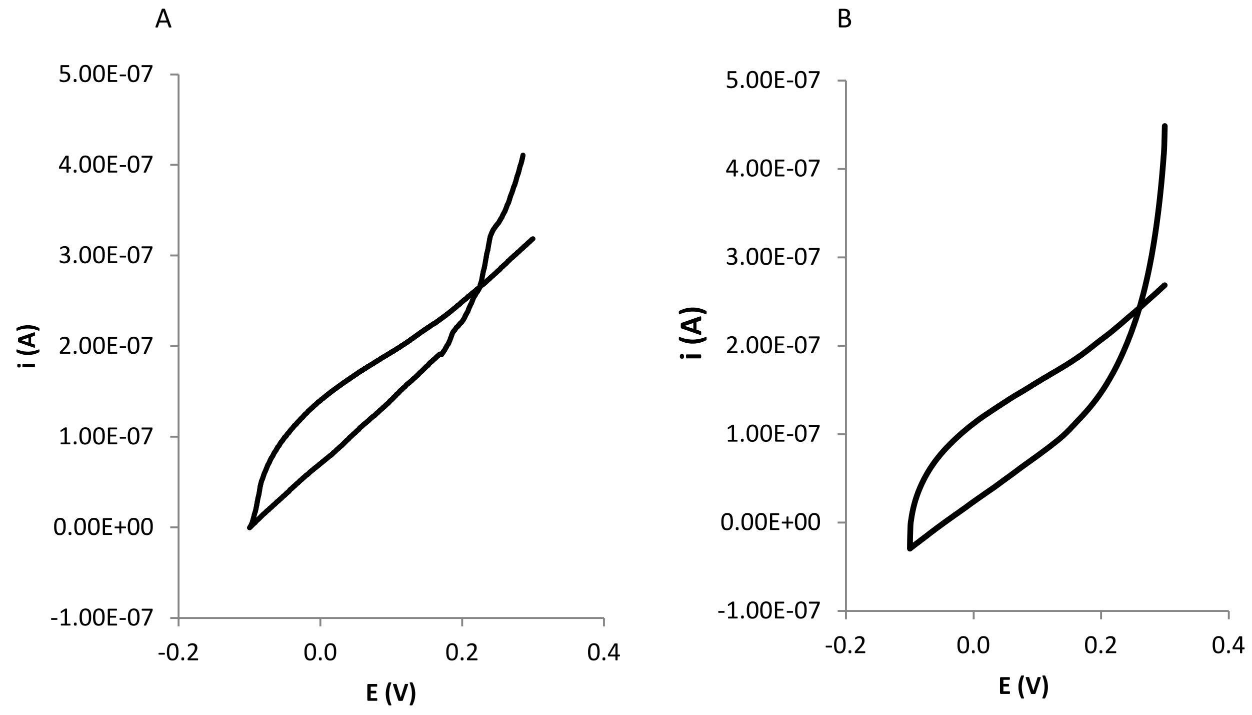

Figure 5S. Cyclic voltammograms recorded in electrolyte consisting of modified DMEM culture medium (modified with FBS/HEPES/antibiotics) at electrodes consisting of gold modified with 3-aminophenylboronic acid in the (A) and (B) absence of RAW cells at 100 mV s^{-1}.

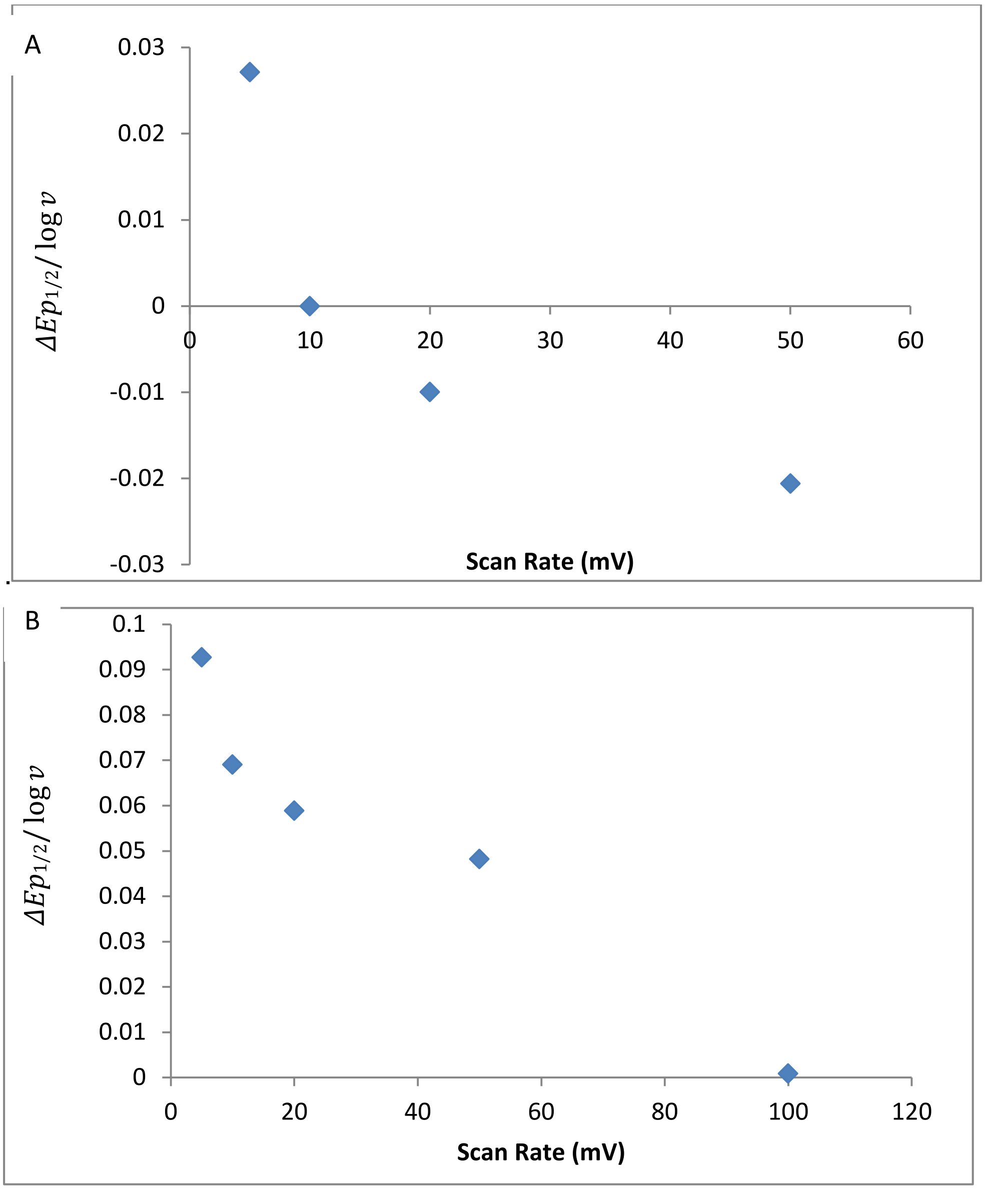

Figure 6S. Rate of shift of peak potential as a function of scan rate for voltammetric peaks A) P1 and B) P3.

Linear sweep voltammograms were logged at surfaces modified with the mercaptobenzoic acid that was subsequently functionalised with a boronic acid. A single peaks were observed at approximately −0.083 V and correspond to the reductive desorption of the adsorbed mercaptobenzoic acid, which proceeds via the 1 electron reduction of the thiol. By integrating the area under the peak we can calculate the surface coverage based on Γ=Q/nFA. Where Γ is the surface coverage in moles cm^2, Q is the charge in coulombs, n is the number of electrons in involved in the rate limiting step, F is the faraday constant and A is the area of the electrode cm^2. This yielded a mean surface coverage of $7.8x10^{-10}$ mol/cm^2 (± n =3). This is close to literature values previously reported for similar structured

SAMs in which a value of 7.7 x 10^{-10} mol/cm^2[9]. Thus this indicates that we have a monolayer coverage and the surfaces are stable.

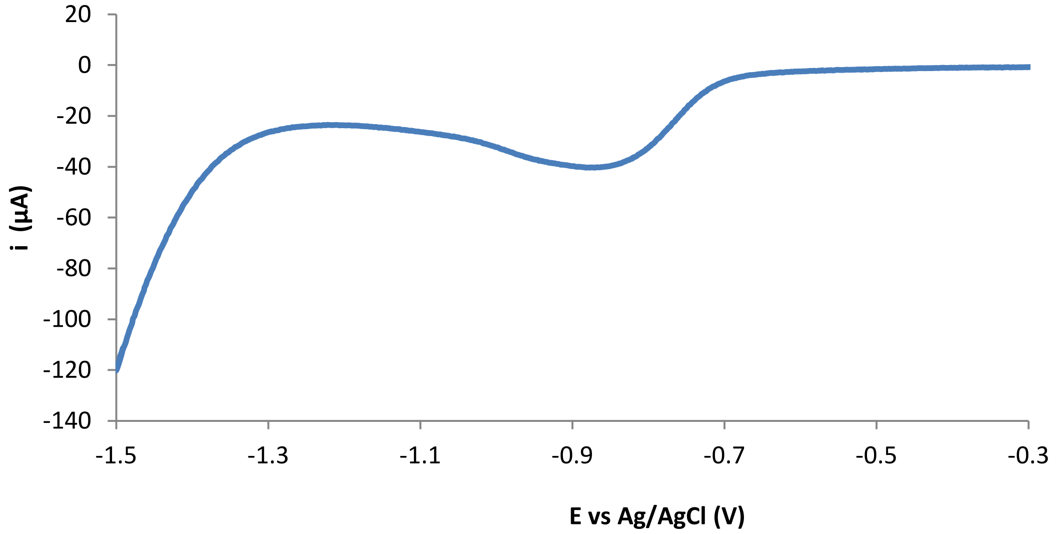

Figure 7S. Typical linear sweep voltammogram for reductive thiol desorption obtained for boronic acid modified surfaces after incubation with cells for 3 hours at a scan rate of100 mV s^{-1}.

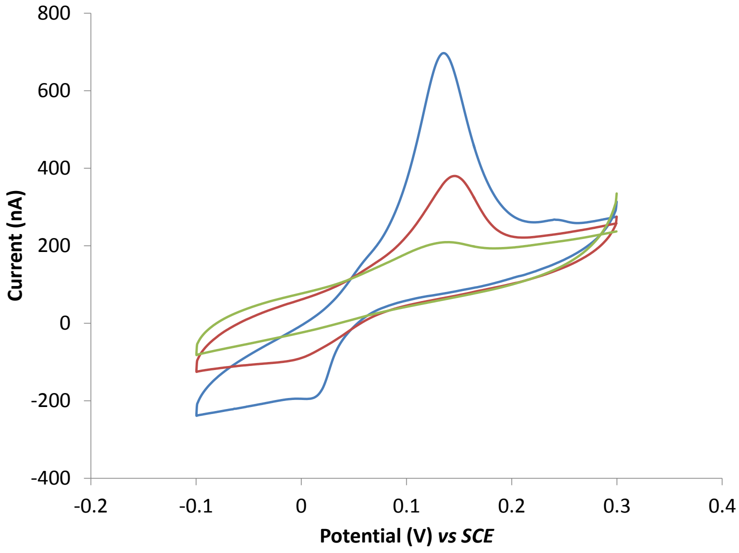

Figure 8S. Consecutive cyclic voltammograms (blue) first, (red) second and (green) third cycle recorded at a working electrode consisting of BA-SAM with macrophage cells attached. The electrolyte was 50 mM PBS with 10% ethanol added as a toxin.

References.

[1] D. Barriet, C. M. Yam, O. E. Shmakova, A. C. Jamison, T. R. Lee, *Langmuir* **2007**, *23*, 8866-8875.

[2] C. Vahlberg, M. Linares, P. Norman, K. Uvdal, *J. Phys. Chem. C* **2011**, *116*, 796-806.

[3] X. Zhong, H.-J. Bai, J.-J. Xu, H.-Y. Chen, Y.-H. Zhu, *Adv. Fun. Mat.* **2010**, *20*, 992-999.

[4] A. Pranzetti, S. Mieszkin, P. Iqbal, F. J. Rawson, M. E. Callow, J. A. Callow, P. Koelsch, J. A. Preece, P. M. Mendes, *Advanced Materials* **2013**, *25*, 2181-2185.

[5] L. Fialkow, Y. Wang, G. P. Downey, *Free Radic. Biol. Med.* **2007**, *42*, 153-164.

[6] W. Dröge, *Physiol. Rev.* **2002**, *82*, 47-95.

[7] C. Bogdan, M. Röllinghoff, A. Diefenbach, *Curr. Opin. Immunol.* **2000**, *12*, 64-76.

[8] J. Zielonka, A. Sikora, M. Hardy, J. Joseph, B. P. Dranka, B. Kalyanaraman, *Chemical Research in Toxicology* **2012**, *25*, 1793-1799.

[9] Y.-T. Tao, C.-C. Wu, J.-Y. Eu, W.-L. Lin, K.-C. Wu, C.-h. Chen, *Langmuir* **1997**, *13*, 4018-4023.
